# Hyperkalemia Recurrence and Its Association With Race and Ethnicity in United States Veterans: A Retrospective Cohort Study

**DOI:** 10.7759/cureus.59003

**Published:** 2024-04-25

**Authors:** Rebecca S Ahdoot, Jui-Ting Hsiung, Abiy Agiro, Yasmin G Brahmbhatt, Kerry Cooper, Souhiela Fawaz, Laura Westfall, Keith C Norris, Kamyar Kalantar-Zadeh, Elani Streja

**Affiliations:** 1 Department of Medicine, Harold Simmons Center for Kidney Disease Research and Epidemiology, University of California Irvine, Orange, USA; 2 Department of Research, Tibor Rubin VA Medical Center, Long Beach, USA; 3 Department of Medical Affairs, AstraZeneca, Wilmington, USA; 4 Department of Medicine, University of California Los Angeles, Los Angeles, USA; 5 Department of Medicine, Harbor-UCLA Medical Center, Torrance, USA

**Keywords:** black, hispanic, risk factors, retrospective study, recurrence, ethnicity, race, hyperkalemia, outcomes, potassium

## Abstract

Introduction: Information on whether race and ethnicity are associated with a greater risk of recurrent hyperkalemia is limited. The aim of this study was to examine the association between race or ethnicity and recurrent hyperkalemia in a population of US veterans.

Methods: This retrospective study used the US Veterans Affairs database to identify adults (aged ≥18 years) with at least one serum potassium measurement during the study period who ever experienced hyperkalemia (serum potassium > 5.0 mmol/L). The proportion of patients with hyperkalemia recurrence (≥1 subsequent event) within one year was determined for different race and ethnicity groups. The association between patient race and ethnicity and the risk of hyperkalemia recurrence within one year after the index hyperkalemia event was analyzed using competing risk regression.

Results: Among a total of 1,493,539 veterans with incident hyperkalemia (median age (interquartile range): 61.0 years (54.0, 71.0)), recurrence within one year occurred in 19.1% of Black, 16.0% of Native Hawaiian/other Pacific Islander, 15.1% of White, 14.9% of American Indian/Alaska Native, and 13.1% of Asian patient groups. Recurrent hyperkalemia occurred in 18.1% of Hispanic and 15.6% of non-Hispanic patient groups. In a fully-adjusted regression model, recurrent hyperkalemia risk was significantly higher in Black versus White patient groups (subhazard ratio (sHR), 1.17; 95% confidence interval (CI), 1.16-1.19; p< 0.0001) and in Hispanic versus non-Hispanic patient groups (sHR, 1.30; 95% CI, 1.28-1.33; p< 0.0001).

Discussion/Conclusion: Among US veterans with incident hyperkalemia, the risk of recurrent hyperkalemia was higher in Black and Hispanic patient groups. This information may be useful for health system screenings to risk stratify patient groups and both guide the frequency of serum potassium monitoring and better understand the root causes of group differences.

## Introduction

Hyperkalemia, defined as abnormally elevated serum potassium (K^+^) levels of >5.0 mmol/L, can cause severe cardiac arrhythmias and increase mortality [1,2]. Hyperkalemia can also be associated with peripheral neuropathy and is a common manifestation of type 4 renal tubular acidosis [2].

The risk of hyperkalemia is increased in patients with comorbidities, particularly in those with chronic kidney disease (CKD), diabetes, or heart failure (HF) [3-5]. Renin-angiotensin-aldosterone system (RAAS) inhibitors, commonly used in the management of hypertension, proteinuric CKD, and secondary prevention of cardiovascular disease, also pose an increased risk of hyperkalemia. This risk is particularly notable in patients with a low estimated glomerular filtration rate (eGFR; <30 mL/min/1.73 m^2^), diabetes, or congestive HF, and in those taking K^+^-sparing diuretics or with high serum K^+^ levels prior to RAAS inhibitor initiation [6]. Other factors that have been associated with an increased risk of hyperkalemia include advanced age, male sex, hospitalizations, and beta-blocker use [7-10].

The association between other patient demographic factors, such as race and ethnicity, and a greater risk of hyperkalemia has been studied. Racial and ethnic differences could be related to group-level differences in socioeconomic status, co-morbidity and/or medication profile, dietary intake, and more. In one study of patients with diabetes, hyperkalemia was more prevalent in White patients than in patients of other races, whereas hypokalemia was more prevalent in Black patients [10]. In a study of patients on hemodialysis, Black patients had lower odds of hyperkalemia and higher odds of hypokalemia compared with White patients; whereas, Hispanic patients had higher odds of hyperkalemia and lower odds of hypokalemia [11]. These studies suggest that differences in K^+^ tolerance, homeostasis, and nutrition may underlie these differences. There may be additional health inequities and social determinants of health, for which race and ethnicity act as a proxy, that may further explain these differences. Currently, there is limited information on how factors such as race and ethnicity are associated with the risk of recurrent hyperkalemia. Among patients who had already experienced hyperkalemia, more information on the root causes of demographic risk factors for recurrent hyperkalemia could guide decisions regarding the frequency of serum K^+^ monitoring, which may minimize unnecessary testing and identify patients who may require more frequent screening to avoid hyperkalemia-related complications.

Although this study does not advocate for the use of race in clinical algorithms and medical decision-making, the aim of this study was to begin to examine the potential relationship between racial or ethnic groups and recurrent hyperkalemia among US veterans to determine whether these factors are independently associated and to commence the discussion on the underlying socioecological causes related to race and ethnicity that may explain these associations.

## Materials and methods

Study design and objectives

The aim of this retrospective cohort study was to describe the rates of hyperkalemia recurrence within one year after the index hyperkalemia event among different racial and ethnic groups and to examine whether race or ethnicity was independently associated with a greater risk of recurrent hyperkalemia. The data source was the United States (US) Veterans Affairs (VA) database, which includes US veterans or persons who served in active military, naval, air, or space service and who were discharged or released under conditions other than dishonorable according to title 38 of the US federal code of regulations [12]. The database was accessed through VA Informatics and Computing Infrastructure servers with special permissions by VA investigators and research staff. Race and ethnicity were self-reported.

The overall population of this database and eligibility criteria have previously been described for an analysis of the relationship between liver disease and hyperkalemia recurrence [13]. Briefly, patients were required to be aged ≥18 years and to have ≥1 serum K^+^ measurement of >5.0 mmol/L between January 2004 and December 2018. As in the earlier study [13], hyperkalemia recurrence was defined as having a serum K^+^ lab value level >5.0 mmol/L that was measured ≥7 days after the index event and following ≥1 serum K^+^ lab value level ≤5.0 mmol/L post-index (i.e., there was at least one serum K^+^ ≤5.0 mmol/L between the index hyperkalemia event and the hyperkalemia recurrence).

Data for this retrospective observational study were extracted from the VA Data Corporate Warehouse. The dataset includes information on patient demographics, medical inpatient and outpatient visits with International Classification of Diseases (ICD) 9 and 10 codes, lab values for potassium and other measurements obtained from the VA Decision Support System, and vital status. eGFR was calculated via serum creatinine values using the 2012 CKD-EPI (Chronic Kidney Disease Epidemiology Collaboration) equation, which was the most commonly used calculation during the study period.

The study was considered exempt from Institutional Review Board approval as dictated by Title 45 Code of Federal Regulations, part 46 of the US, specifically 45 CFR 46.101(b)(4). In accordance with the Health Insurance Portability and Accountability Act Privacy Rule, disclosed data were considered anonymized per 45 CFR 164.506(d)(2)(ii)(B) through the “Expert Determination” method; no individual patient information was reported.

Statistical analysis

The statistical analyses conducted in this study have previously been described [13]. No formal hypothesis testing was conducted. Patient characteristics were described on the basis of race and ethnicity, using means and standard deviations (SDs), medians and interquartile ranges, or counts and percentages, as appropriate. The possible association between race and ethnicity and hyperkalemia recurrence within one year was analyzed with a Fine and Gray competing risk regression model using the other patient characteristics and comorbidities listed in Table 1 as covariates. Patients were followed for up to one year after the index hyperkalemia event with hyperkalemia recurrence as the main outcome and all-cause mortality as the competing event. Subhazard ratios (sHRs) and 95% confidence intervals (CIs) were ascertained by race and ethnicity. Covariates were selected a priori on the basis of clinical relevance and had been evaluated jointly for their association with the outcome in a prior study [13]. Charts were used to show the proportion of patients (by race and ethnicity) on RAAS inhibitors before and after the index hyperkalemia event and the proportion (by race and ethnicity) of hyperkalemia recurrence within one year of the index event. All analyses were conducted using SAS software, version 9.4 (SAS Institute, Cary, United States).

**Table 1:** Patient demographics and clinical characteristics stratified by race and ethnicity AIDS: Acquired immunodeficiency syndrome; CCI: Charlson Comorbidity Index; COPD: Chronic obstructive pulmonary disease; eGFR: Estimated glomerular filtration rate; HIV: Human immunodeficiency virus; IQR: Interquartile range; K+: Potassium; RAAS: Renin-angiotensin-aldosterone system; SD: Standard deviation

	White (n = 1,184,008)	Black (n= 213,725)	Asian (n= 7,832)	American Indian or Alaska Native (n= 11,741)	Native Hawaiian or Other Pacific Islander (n= 13,747)	Other Race (n= 62,486)	Hispanic or Latino (n= 77,069)	Not Hispanic or Latino (n= 1,364,656)	Other ethnicity (n= 51,814)
Age (years), mean ± SD	62.4 ± 12.8	57.1 ± 12.6	56.2 ± 16.1	57.7 ± 12.5	60.4 ± 13.4	61.0 ± 13.3	58.1 ± 14.7	61.6 ± 12.8	62.5 ± 13.0
Sex, n (%)									
Male	1,141,869 (96.4)	200,709 (93.9)	7,288 (93.1)	11,041 (94.0)	13,038 (94.8)	59,765 (95.6)	73,806 (95.8)	1,310,235 (96.0)	49,669 (95.9)
Female	42,139 (3.6)	13,016 (6.1)	544 (6.9)	700 (6.0)	709 (5.2)	2,721 (4.4)	3,263 (4.2)	54,421 (4.0)	2,145 (4.1)
Ethnicity, n (%)									
Hispanic or Latino	61,420 (5.2)	4,467 (2.1)	300 (3.8)	1,149 (9.8)	1,679 (12.2)	8,054 (12.9)	77,069 (100.0)	0 (0.0)	0 (0.0)
Not Hispanic or Latino	1,104,390 (93.3)	206,393 (96.6)	7,401 (94.5)	10,384 (88.4)	11,692 (85.1)	24,396 (39.0)	0 (0.0)	1,364,656 (100.0)	0 (0.0)
Unknown/declined to answer	18,198 (1.5)	2,865 (1.3)	131 (1.7)	208 (1.8)	376 (2.7)	30,036 (48.1)	0 (0.0)	0 (0.0)	51,814 (100.0)
Race, n (%)									
White	1,184,008 (100.0)	0 (0.0)	0 (0.0)	0 (0.0)	0 (0.0)	0 (0.0)	61,420 (79.7)	1,104,390 (80.9)	18,198 (35.1)
Black	0 (0.0)	213,725 (100.0)	0 (0.0)	0 (0.0)	0 (0.0)	0 (0.0)	4,467 (5.8)	206,393 (15.1)	2,865 (5.5)
Asian	0 (0.0)	0 (0.0)	7,832 (100.0)	0 (0.0)	0 (0.0)	0 (0.0)	300 (0.4)	7,401 (0.5)	131 (0.3)
American Indian or Alaska Native	0 (0.0)	0 (0.0)	0 (0.0)	11,741 (100.0)	0 (0.0)	0 (0.0)	1,149 (1.5)	10,384 (0.8)	208 (0.4)
Native Hawaiian or Other Pacific Islander	0 (0.0)	0 (0.0)	0 (0.0)	0 (0.0)	13,747 (100.0)	0 (0.0)	1,679 (2.2)	11,692 (0.9)	376 (0.7)
Unknown/declined to answer	0 (0.0)	0 (0.0)	0 (0.0)	0 (0.0)	0 (0.0)	62,486 (100.0)	8,054 (10.5)	24,396 (1.8)	30,036 (58.0)
Index eGFR (mL/min/1.73 m^2^), mean ± SD	67.1 ± 23.9	69.6 ± 31.0	70.5 ± 26.7	69.6 ± 25.4	66.5 ± 24.5	67.1 ± 24.9	72.9 ± 25.8	67.2 ± 25.0	65.8 ± 24.8
Index sK^+^ (mmol/L), mean ± SD	5.3 ± 0.3	5.4 ± 0.4	5.3 ± 0.3	5.3 ± 0.3	5.3 ± 0.3	5.3 ± 0.3	5.3 ± 0.3	5.3 ± 0.3	5.3 ± 0.3
CCI score, median (IQR)	1 (0, 3)	2 (0, 4)	1 (0, 2)	1 (0, 3)	1 (0, 3)	1 (0, 3)	1 (0, 2)	1 (0, 3)	1 (0, 3)
Comorbidities, n (%)									
Diabetes	381,057 (32.2)	80,879 (37.8)	2,676 (34.2)	4,465 (38.0)	5,083 (37.0)	20,671 (33.1)	28,419 (36.9)	449,509 (32.9)	16,903 (32.6)
COPD	206,579 (17.4)	32,190 (15.1)	634 (8.1)	1,921 (16.4)	1,985 (14.4)	9,965 (15.9)	7,157 (9.3)	236,812 (17.4)	9,305 (18.0)
Kidney disease	166,940 (14.1)	54,583 (25.5)	1,093 (14.0)	1,918 (16.3)	2,133 (15.5)	9,708 (15.5)	10,071 (13.1)	217,838 (16.0)	8,466 (16.3)
Cancer	149,569 (12.6)	35,232 (16.5)	633 (8.1)	1,248 (10.6)	1,582 (11.5)	7,942 (12.7)	8,013 (10.4)	181,050 (13.3)	7,143 (13.8)
Congestive heart failure	133,842 (11.3)	29,750 (13.9)	619 (7.9)	1,283 (10.9)	1,465 (10.7)	7,270 (11.6)	5,806 (7.5)	161,746 (11.9)	6,677 (12.9)
Peripheral vascular disease	102,485 (8.7)	16,880 (7.9)	337 (4.3)	845 (7.2)	1,016 (7.4)	5,046 (8.1)	4,177 (5.4)	117,785 (8.6)	4,647 (9.0)
Cerebrovascular disease	74,089 (6.3)	16,692 (7.8)	363 (4.6)	672 (5.7)	817 (5.9)	4,010 (6.4)	4,038 (5.2)	88,883 (6.5)	3,722 (7.2)
Myocardial infarction	62,518 (5.3)	9,765 (4.6)	267 (3.4)	585 (5.0)	674 (4.9)	3,221 (5.2)	2,890 (3.8)	71,312 (5.2)	2,828 (5.5)
Liver disease	48,647 (4.1)	18,454 (8.6)	248 (3.2)	726 (6.2)	638 (4.6)	3,077 (4.9)	4,927 (6.4)	64,410 (4.7)	2,453 (4.7)
Dementia	38,356 (3.2)	9,973 (4.7)	200 (2.6)	272 (2.3)	440 (3.2)	2,157 (3.5)	2,836 (3.7)	46,516 (3.4)	2,046 (4.0)
Rheumatologic disease	18,196 (1.5)	2,949 (1.4)	77 (1.0)	200 (1.7)	206 (1.5)	947 (1.5)	856 (1.1)	20,931 (1.5)	788 (1.5)
Peptic ulcer disease	12,842 (1.1)	3,248 (1.5)	89 (1.1)	123 (1.0)	147 (1.1)	731 (1.2)	883 (1.2)	15,650 (1.2)	647 (1.3)
Hemiplegia/paraplegia	11,666 (1.0)	4,163 (1.9)	84 (1.1)	136 (1.2)	153 (1.1)	838 (1.3)	868 (1.1)	15,450 (1.1)	722 (1.4)
AIDS/HIV	3,644 (0.3)	4,663 (2.2)	21 (0.3)	60 (0.5)	67 (0.5)	265 (0.4)	621 (0.8)	7,862 (0.6)	237 (0.5)
Concomitant medications, n (%)									
RAAS inhibitor	570,256 (48.2)	102,913 (48.2)	3,808 (48.6)	5,623 (47.9)	6,637 (48.3)	30,006 (48.0)	37,136 (48.2)	657,171 (48.2)	24,936 (48.1)
Beta-blocker	457,090 (38.6)	82,523 (38.6)	3,067 (39.2)	4,503 (38.4)	5,365 (39.0)	24,131 (38.6)	29,709 (38.6)	527,019 (38.6)	19,951 (38.5)
Calcium-channel blocker	258,099 (21.8)	46,840 (21.9)	1,659 (21.2)	2,612 (22.2)	3,073 (22.4)	13,698 (21.9)	16,575 (21.5)	298,114 (21.9)	11,292 (21.8)
Thiazide	222,010 (18.8)	40,161 (18.8)	1,419 (18.1)	2,211 (18.8)	2,621 (19.1)	11,781 (18.9)	14,262 (18.5)	256,216 (18.8)	9,725 (18.8)
Loop diuretic	204,979 (17.3)	36,953 (17.3)	1,307 (16.7)	2,031 (17.3)	2,424 (17.6)	10,858 (17.4)	13,103 (17.0)	236,456 (17.3)	8,993 (17.4)
Alpha-blocker	129,831 (11.0)	23,543 (11.0)	843 (10.8)	1,288 (11.0)	1,525 (11.1)	6,924 (11.1)	8,341 (10.8)	149,848 (11.0)	5,765 (11.1)
RAAS inhibitor + diuretic	76,953 (6.5)	13,809 (6.5)	505 (6.4)	768 (6.5)	907 (6.6)	4,043 (6.5)	4,895 (6.4)	88,678 (6.5)	3,412 (6.6)
K^+^-sparing diuretic	76,204 (6.4)	13,640 (6.4)	502 (6.4)	760 (6.5)	902 (6.6)	4,003 (6.4)	4,849 (6.3)	87,775 (6.4)	3,387 (6.5)
Vasodilator	39,037 (3.3)	7,204 (3.4)	290 (3.7)	394 (3.4)	485 (3.5)	2,105 (3.4)	2,451 (3.2)	45,270 (3.3)	1,794 (3.5)

## Results

Patients

Among the 9,894,683 US veterans with at least one serum K^+^ measurement between 0.5 mmol/L and 8.0 mmol/L during the study period, 1,493,539 experienced an index hyperkalemia event and were included in this study [13].

The patient demographics and clinical characteristics of the total population have previously been described [13]. Baseline characteristics are presented for each racial and ethnic group within the overall population in Table 1. The median age (interquartile range) of the overall population was 61.0 (54.0, 71.0) years, reflecting that US veterans are an elderly population.

The study population consisted mostly of White patients (n = 1,184,008; 79.3%), with smaller proportions of patients of other races: 14.3% Black patients (n = 213,725), 0.9% Native Hawaiian or other Pacific Islander patients (n = 13,747), 0.8% American Indian or Alaska Native patients (n = 11,741), and 0.5% Asian patients (n = 7,832). The remaining 4.2% of patients (n = 62,486) reported having other races. The study included 77,069 patients with Hispanic or Latino ethnicity; most patients did not have Hispanic or Latino ethnicity (n = 1,364,656; 91.4%) and 51,814 patients (3.5%) reported having other ethnicity. The proportion of patients with Hispanic or Latino ethnicity was higher among Native Hawaiian or other Pacific Islander patients (12.2%) and American Indian or Alaska Native patients (9.8%) than among White (5.2%), Asian (3.8%), or Black (2.1%) patient groups.

**Figure 1:**
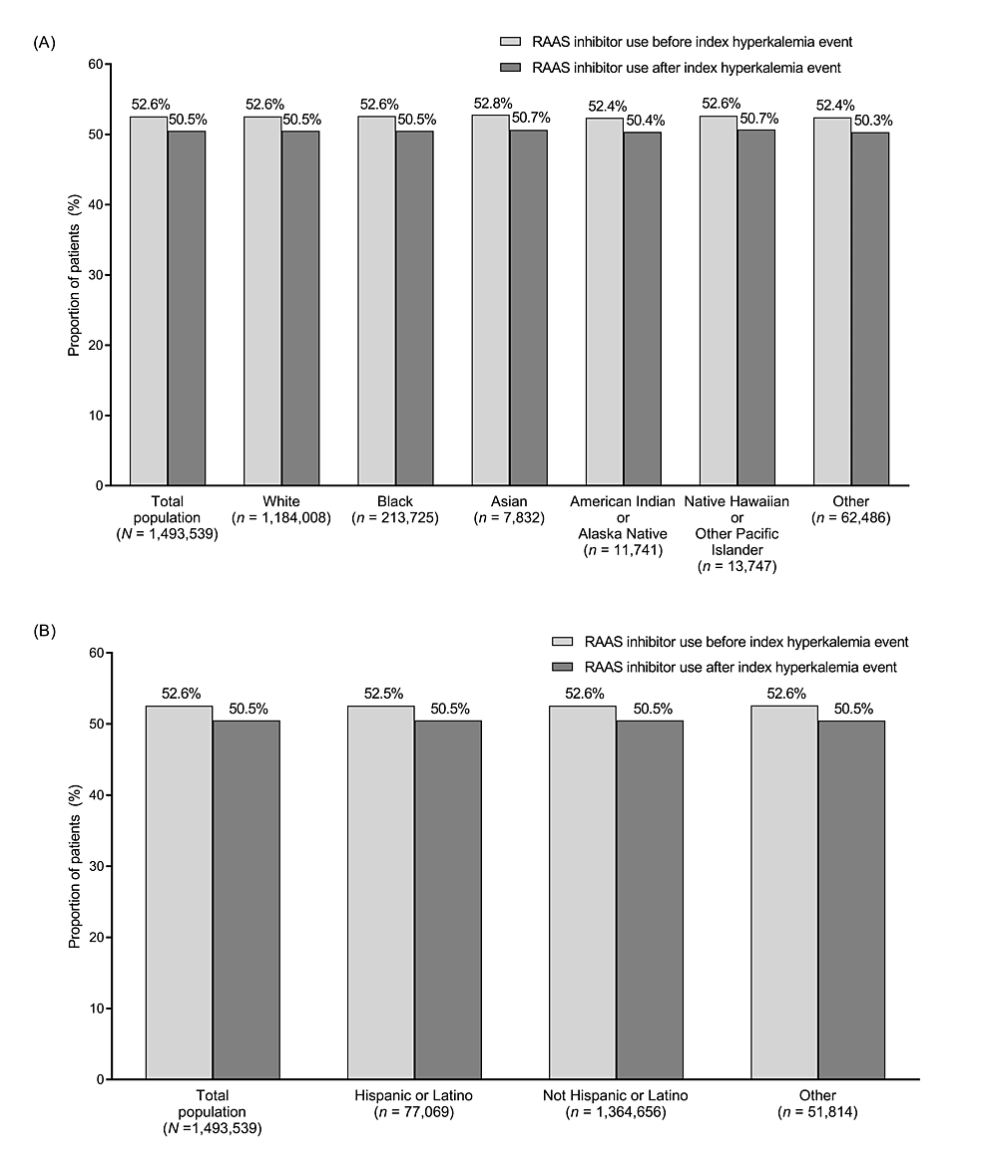
Proportion of patients taking renin-angiotensin-aldosterone system (RAAS) inhibitors before and after the index hyperkalemia event overall and by (A) race and (B) ethnicity RAAS: Renin-angiotensin-aldosterone system

The use of concomitant medications was similar across the different racial and ethnic groups. In particular, the proportion of patients receiving concomitant RAAS inhibitor therapy was similar between races, ranging from 52.4% to 52.8% before the index hyperkalemia event and from 50.3% to 50.7% after the index event (Figure 1A). The proportion of patients receiving concomitant RAAS inhibitor therapy was also similar between different ethnicities (Figure 1B).

Hyperkalemia recurrence

**Figure 2:**
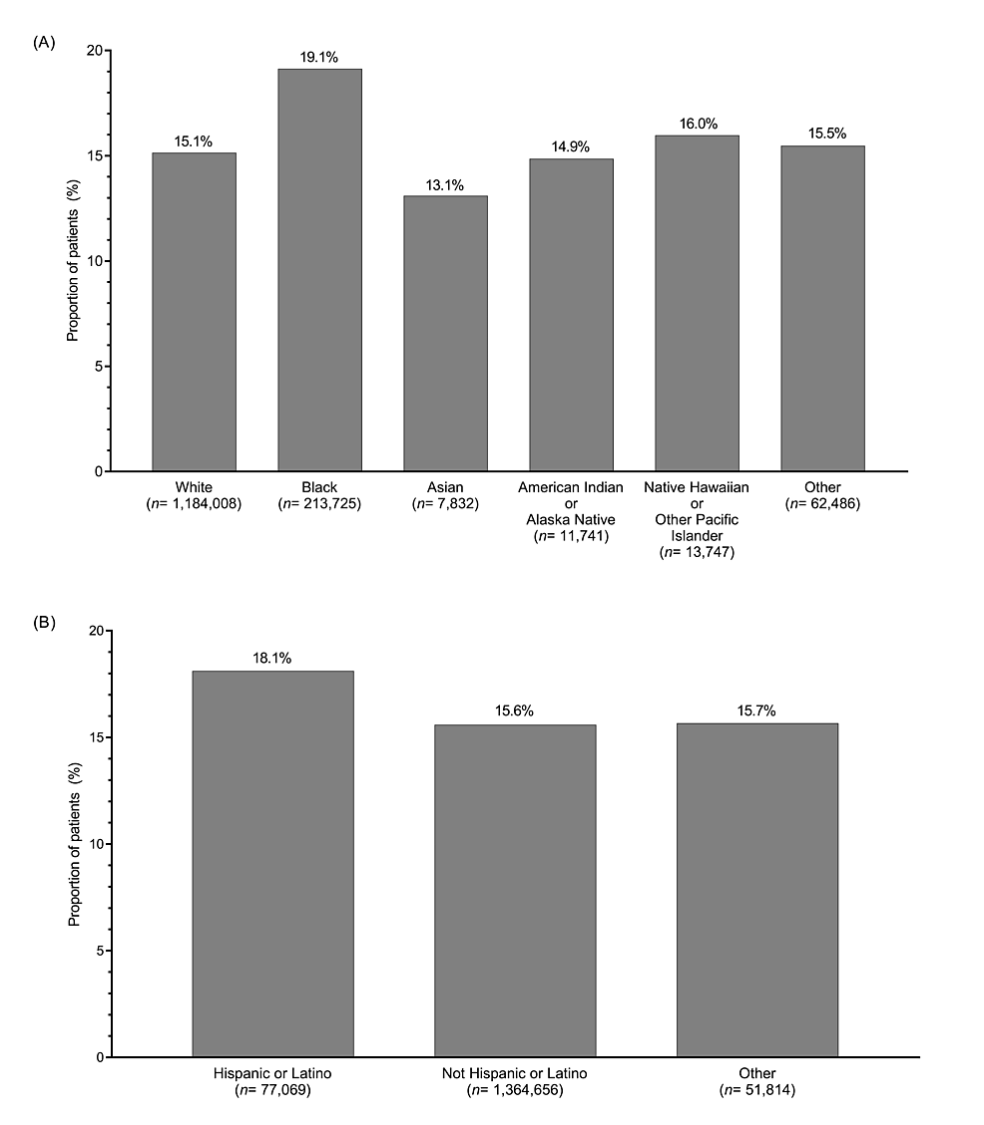
Proportion of patients with recurrent hyperkalemia within one year after the index hyperkalemia event by (A) race and (B) ethnicity

Hyperkalemia recurrence within one year after the index hyperkalemia event occurred in 234,807 patients (15.7%) in the total population [13]. When analyzed by race, the proportion of patients with hyperkalemia recurrence within one year was highest among Black patients (19.1%; n= 40,879), followed by Native Hawaiian or other Pacific Islander patients (16.0%; n= 2,196) (Figure 2A). Asian patients had the lowest rate of recurrent hyperkalemia (13.1%;n= 1,026) (Figure 2A). In the analysis of hyperkalemia recurrence by ethnicity, the proportion of patients with recurrent hyperkalemia within one year was higher among patients with Hispanic or Latino ethnicity (18.1%; n = 13,961) than among those with non-Hispanic or Latino ethnicity (15.6%; n = 212,734) (Figure 2B).

Characteristics associated with hyperkalemia recurrence

In the competing risk regression analysis, the risk of recurrent hyperkalemia within one year after the index hyperkalemia event was significantly higher in Black versus White patients (by 17%; sHR, 1.17; 95% CI, 1.16-1.19; p< 0.0001) and in patients with Hispanic or Latino ethnicity versus those without Hispanic or Latino ethnicity (by 30%; sHR, 1.30; 95% CI, 1.28-1.33; p< 0.0001) (Table 2). The risk of hyperkalemia recurrence was significantly lower in American Indian or Alaska Native patients (sHR, 0.94; 95% CI, 0.90-0.99; p= 0.0135) compared with White patients.

**Table 2:** Regression analysis of race and ethnicity factors for recurrent hyperkalemia within one year of the index hyperkalemia event CI: Confidence interval

	Subhazard ratio from competing risk regression (95% CI)	P-value
Race (vs. White)		
American Indian or Alaska Native	0.94 (0.90-0.99)	0.0135
Asian	0.94 (0.89-1.00)	0.0551
Black	1.17 (1.16-1.19)	<0.0001
Native Hawaiian or Pacific Islander	1.01 (0.97-1.00)	0.5606
Unknown	0.98 (0.95-1.00)	0.0605
Ethnicity (vs. non-Hispanic)		
Hispanic or Latino	1.30 (1.28-1.33)	<0.0001
Unknown	1.02 (0.99-1.04)	0.2668

## Discussion

In this large retrospective cohort study of 1,493,539 US veterans with incident hyperkalemia, the proportion of patients with recurrent hyperkalemia within one year was highest among Black patients (19.1%) and those of Hispanic or Latino ethnicity (18.1%), whereas the rate of hyperkalemia recurrence was lowest in Asian patients (13.1%). Race and ethnicity were both significantly associated with hyperkalemia recurrence within one year after the index hyperkalemia event, with the risk being significantly higher in Black versus White patients and patients with Hispanic or Latino ethnicity versus those without, and significantly lower in American Indian or Alaska Native versus White patients. The observed racial and ethnic differences in hyperkalemia recurrence were independent of medications typically associated with hyperkalemia, such as RAAS inhibitors and K^+^-sparing diuretics.

In previous studies, independent predictors of hyperkalemia risk have included older age, male sex, decreased eGFR, inpatient hospitalization, the presence of comorbidities (including diabetes, CKD, and HF), and RAAS inhibitor use [7-10,14]. With regard to race and ethnicity, previous studies in patients with diabetes (N= 321,856) [10] or in patients undergoing hemodialysis (N= 102,241) [11] indicated that the risk of hyperkalemia was lower in Black versus White patients and higher in Hispanic versus White patients. Furthermore, a study in two large cohorts found that, in addition to a lower risk of hyperkalemia, Black participants had a lower mean serum K^+ ^level and a higher risk of hypokalemia than non-Black participants [15].

In a previous study by our group using the same patient database as the current study, we found a strong association of liver disease with hyperkalemia recurrence [13]. Other characteristics significantly associated with hyperkalemia recurrence in this population included diabetes, congestive HF, and lower eGFR category [13]. To our knowledge, no prior study has looked at predictors of recurrent hyperkalemia among different racial and ethnic groups. Similar to previous studies that looked at predictors of hyperkalemia, our study found that Hispanic ethnicity was associated with a greater risk of recurrent hyperkalemia. However, unlike other studies that only looked at predictors of hyperkalemia, we found that the risk of recurrent hyperkalemia was also higher in Black patients.

Previous data suggest that Black cohorts may better tolerate elevated serum K^+ ^levels than White populations, with hyperkalemia (serum K^+ ^> 5.5 mmol/L) being associated with an increased risk of mortality in White but not Black cohorts [16]. The reasons for differences in recurrent hyperkalemia risk between races and ethnicities are unclear but may be caused, at least in part, by racial differences in K^+ ^homeostasis in Black populations, who as a group have lower urinary potassium excretion and decreased cellular potassium disposal compared with White populations [16,17], though the underlying reasons are unclear. Differences in hyperkalemia risk between Hispanic and non-Hispanic groups of patients may be due to variations in diet between ethnicities, with dietary modifications being more challenging in Hispanic populations because of cultural influences on food choice [11,18]. Additionally, other social and ecologic factors related to race and ethnicity in veterans, which could not be accounted for in our study, could also explain these differences [19]. Of note, Black and Hispanic populations have an increased incidence of CKD compared with White or non-Hispanic populations, with end-stage kidney disease occurring three times more commonly in Black versus White populations and having a 31.4% higher incidence in Hispanic versus non-Hispanic populations [20]. In addition, the prevalence of diabetes is higher in non-Hispanic Black and Hispanic American populations than in those of non-Hispanic White, Asian American, or Alaskan Native race or ethnicity [21]. Given that lower eGFR and diabetes were both associated with an increased risk of hyperkalemia recurrence in the current study, the presence of these comorbidities likely contributed to the observed increase in the risk of recurrent hyperkalemia among Black and Hispanic veterans. Further research is needed to explore the reasons for the racial and ethnic differences in the risk of hyperkalemia recurrence and the possible implications for the clinical management of patients with hyperkalemia.

This study had limitations. The retrospective observational cohort study design means that there may be confounding variables. For instance, we did not have access to information on socioeconomic factors and community-level resources that may influence the prevalence of “blue collar” jobs, nutrition and dietary patterns, volume status, pre-clinical disease severity, use of over-the-counter medications, health behaviors, and other factors that can influence K^+ ^regulation and be differentially distributed across racial and ethnic groups because of structural racism and differential group-level access to health-affirming resources and opportunities. Due to the observational nature of our study, results should be interpreted with caution and no causal associations may be concluded. Furthermore, although our study has found differences in hyperkalemia recurrence with race and ethnicity, we do not advocate for the use of race or ethnicity - which are population-level, unordered latent/proxy social variables - in formulas for individual-level medical decision-making or clinical algorithms, as these would be methodologically inappropriate; instead, our findings can be used for group-level awareness, education, and messaging [19]. In addition, our study population comprised patients who were identified from the US VA health system database, who are mostly older White males with a higher number of comorbidities; therefore, the results of this study may not be generalizable to the overall or non-veteran US populations.

## Conclusions

In this study, the risk of recurrent hyperkalemia was highest in Black and Hispanic patients. This information may be useful for population-level and health system-level screening. It also warrants further studies on the underlying socioecological reasons for racial and ethnic differences in hyperkalemia recurrence and the possible implications for clinical practice, including the frequency of serum K^+ ^monitoring and the prescription of concomitant medications in patients at high risk.
